# Growth, photosynthetic function, and stomatal characteristics of Persian walnut explants *in vitro* under different light spectra

**DOI:** 10.3389/fpls.2023.1292045

**Published:** 2023-11-17

**Authors:** Seyyed Arash Saeedi, Kourosh Vahdati, Saadat Sarikhani, Shirin Dianati Daylami, Maryam Davarzani, Nazim S. Gruda, Sasan Aliniaeifard

**Affiliations:** ^1^Department of Horticulture, College of Aburaihan, University of Tehran, Tehran, Iran; ^2^Department of Horticultural Science, INRES–Institute of Crop Science and Resource Conservation, University of Bonn, Bonn, Germany; ^3^Controlled Environment Agriculture Center (CEAC), College of Agriculture and Natural Resources, University of Tehran, Tehran, Iran

**Keywords:** chlorophyll fluorescence, *in vitro* hardening, LED light, light quality, photosynthetic functionality, micropropagation

## Abstract

Light plays a crucial role in photosynthesis, which is an essential process for plantlets produced during *in vitro* tissue culture practices and *ex vitro* acclimatization. LED lights are an appropriate technology for *in vitro* lighting but their effect on propagation and photosynthesis under *in vitro* condition is not well understood. This study aimed to investigate the impact of different light spectra on growth, photosynthetic functionality, and stomatal characteristics of micropropagated shoots of Persian walnut (cv. Chandler). Tissue-cultured walnut nodal shoots were grown under different light qualities including white, blue, red, far-red, green, combination of red and blue (70:30), combination of red and far-red (70:30), and fluorescent light as the control. Results showed that the best growth and vegetative characteristics of *in vitro* explants of Persian walnut were achieved under combination of red and blue light. The biggest size of stomata was detected under white and blue lights. Red light stimulated stomatal closure, while stomatal opening was induced under blue and white lights. Although the red and far-red light spectra resulted in the formation of elongated explants with more lateral shoots and anthocyanin content, they significantly reduced the photosynthetic functionality. Highest soluble carbohydrate content and maximum quantum yield of photosystem II were detected in explants grown under blue and white light spectra. In conclusion, growing walnut explants under combination of red and blue lights leads to better growth, photosynthesis functionality, and the emergence of functional stomata in *in vitro* explants of Persian walnuts.

## 1 Introduction

In tissue culture practices artificial light sources, including fluorescent lights, high-pressure sodium lights, metal halide lights, and incandescent lights, have been used (Dutta Gupta and Jatothu, 2013; Cavallaro et al., 2022). Fluorescent lights are the most widely used in tissue culture rooms (Economou and Read, 1987). They have limited possibilities to control and adjust the intensity and composition of the light spectra, and these parameters are not stable and change during the growth of tissue culture plants (Darko et al., 2014; Bantis et al., 2016). They have wide wavelength ranges (350 to 750 nm). Such a wide range of wavelengths seems unnecessary, and is of poor quality for plant growth, generating heat in the laboratory (Guiamba et al., 2022; Hamedalla et al., 2022). Furthermore, tissue culture samples cannot be so close to these light sources (Anuchai, 2020). Due to the low intensity of red and far-red lights, they emit little photosynthetically active radiation (PAR= 20-30%) and the wavelength of these lights is usually in the green and blue region (Miler et al., 2019). The use of fluorescent lights in the production of tissue culture plants also incurs significant costs associated with electricity consumption (Kulus and Woźny, 2020). Therefore, for successful commercial micropropagation in plant tissue culture laboratories, an effective light source is needed to improve production efficiency, reduce costs, and improve the quality of tissue culture plants.

The acclimatization process is not only affected by *ex vitro* environment but also by the *in vitro* condition, which is often considered a hardiness process (Hazarika, 2006). Improper development of the photosynthetic system and malfunctioning of stomata during *in vitro* culture may lead to a series of morphological and physiological disorders of plant tissue (Urban et al., 2017). Although tissue-cultured plants may look normal *in vitro*, they are unlikely to be actively photosynthesizing (Askari et al., 2022). This is due to the provision of sucrose as the source of carbon and different environments than *ex vitro*, which disturb the natural development of the photosynthetic apparatus and stomatal responses afterward (Gago et al., 2014). To overcome the shortcomings of tissue culture plant production on a large scale, the development of new environmental control systems in *in vitro* conditions is necessary.

One of the biggest challenges in tissue culture propagation is the high mortality rate of plants when they are moved from the lab to the greenhouse or field (Kumar and Rao, 2012). This is a common issue in horticulture and there is ongoing research on developing effective protocols to ensure the survival of plantlets after *in vitro* propagation (Aliniaeifard et al., 2020). The change in the morphology, anatomy, and physiology of tissue culture-generated plants impose problems for the growth and development of plants in the adaptation stage, as a result, control of the physical and chemical conditions of the *in vitro* environment should be considered in order to facilitate the process of adaptation to new environmental conditions. Successful adaptation of plants grown *in vitro* can improve their growth in *ex vitro* conditions (Pospóšilová et al., 1999).

Proper functionality of stomata plays a vital role in survival of tissue-cultured plants. This helps maintain the leaf’s water levels and enhances photosynthetic performance (Asayesh et al., 2017a). Improvements in this area include increased chlorophyll production, effective electron transport within the chloroplasts, better photochemical efficiency, and a higher net rate of photosynthesis (Haisel et al., 2004). The *in vitro* plantlets are grown in different CO_2_ concentrations compared to the natural environments, greenhouse, and field. The existence of such conditions in the environment of *in vitro* cultures eventually leads to the formation of plants with unusual morphology, anatomy, and physiology, which often have poor photosynthetic efficiency, stomatal dysfunction, and reduced cuticle development (Nejad and Van Meeteren, 2005; Aliniaeifard and van Meeteren, 2014; Asayesh et al., 2017b; Vahdati et al., 2017; Asayesh et al., 2021).

Due to the addition of sugar to the medium, photosynthesis in tissue-cultured explants decreases, however, the removal of sucrose is detrimental to growth of tissue culture plantlets (Bidabadi and Jain, 2020). It is wrongly claimed that due to the presence of sugar in the medium, *in vitro*, plants do not need photosynthesis. It has been shown that the removal of light and CO_2_ as the two main inputs of photosynthesis is harmful for the growth of tissue cultured plants (Askari et al., 2022). Adaptation of plantlets after *in vitro* growth needs practices such as reducing RH, increasing light level, and CO_2_ concentration, or reducing the osmotic potential of the culture medium (Aliniaeifard et al., 2020). Meanwhile, the stomata of tissue-cultured plants are abnormal with wide openings and malfunctioning to close completely in response to external closing factors. This leads to the loss of a large amount of water in the plant tissue during the transition stage (Fabbri et al., 1986). Due to these disorders, tissue-cultured plants are susceptible to wilting and desiccation after being transferred to natural conditions or an environment with low relative humidity. The direct transfer of these plants without adaptation would result in failure in production (Gribaudo et al., 2001; Fanourakis et al., 2013; Aliniaeifard et al., 2020).

In most tissue culture plants, which are normally grown at high relative humidity, stomatal function is disrupted and the opening of stomata in these plants causes relatively intense stress in the first few hours after transfer to *ex vitro* environment (Majada et al., 1998). The stomatal structure of tissue culture plants is also different from that of plants grown in the greenhouse (Asayesh et al., 2017a). In response to external stimuli, the stomata of tissue culture plants often fail to close completely (Polivanova and Bedarev, 2022). Tissue culture plants have round and open stomata with higher stomatal density and index compared to adapted greenhouse plants. On the other hand, their water loss rate is significantly higher than that of adapted and greenhouse plants (Joshi et al., 2006; Bertolino et al., 2019). The high and uneven stomatal density and variation in stomatal size may be related to the rapid wilting of leaves under high stress.

Little information is available on the photosynthetic functionality of Persian walnut explants in particular, although photosynthesis is important for plant growth and development. An important step in the process of walnut propagation is the *in vitro* hardening of walnut tissue culture plants (Vahdati et al., 2021). *In vitro*, hardening is a process by which plants grown in tissue culture are adapted to *ex vitro* field conditions (McCartan et al., 2004). The process involves the gradual overcoming of abnormalities and the adaptation to the *ex vitro* field conditions (McCartan et al., 2004). A *in vitro* Desiccation is the result of rapid water loss, which can lead to the death of plants immediately after transplantation. Therefore, the development of efficient stomata in walnut tissue cultured plants is of vital importance.

Morphogenesis and its related aspects are mainly regulated by various photoreceptors that are activated by photons in the range of blue, red, and far-red light spectra (Griffin and Toledo-Ortiz, 2022). In recent years, light-emitting diodes (LEDs) have been used as a potential alternative light source for the growth and development of tissue culture plants. LEDs allow manipulation of the light’s quality suitable for plants’ needs (Trivellini et al., 2023). Compared to conventional systems, LED lighting systems have unique advantages such as durability, small size, long operating lifetime, relatively cool emitting surface, a linear photon output with the electrical input current, and the ability to control spectral composition (Brown et al., 1995). Light is essential for photosynthesis, but it can also be detrimental if photosynthesis is inhibited by desiccation (McCartan et al., 2004). Since tissue culture depends entirely on artificial light sources for illumination, the effect of the light spectrum on the growth, stomatal function, and photosynthetic functionality is a matter of great interest.

Micropropagation of all species of *Juglans* genus is very difficult, but it is widely used for the mass propagation of Persian walnuts. Raising the proliferation rate to a high level is one of the necessities of commercial micropropagation (Julian et al., 2020). Only some laboratories around the world have commercial micropropagation capacity. However, plantlet adaptation is considered as a challenging stage in walnut micropropagation (Litz et al., 2020). Because the light spectrum strongly influences the stomata and its reactions, therefore, in this research, it was assumed that different light spectra would alter stomatal morphology *in vitro*. Photosynthesis is directly affected by the light spectrum, and since photosynthesis provides the carbohydrates needed for plant growth, it was assumed that different light spectra could influence the photosynthetic performance of tissue-cultured explants. However, there is insufficient information on how various light spectra impact growth, stomatal morphology and anatomy, and the biophysical characteristics of photosynthesis. To address this gap, we conducted a study exposing walnut tissue culture explants to different light spectra during their proliferation stage. Our objective was to induce functional stomata and investigate the growth, photosynthetic functionality, and stomatal characteristics of the walnut explants under *in vitro* application of different light spectra.

## 2 Materials and methods

### 2.1 Propagation of walnut *in vitro*

Micro-propagated shoots of Persian walnut (cv. Chandler) were used in the proliferation stage, which were transferred to a fresh culture medium once every 3 to 4 weeks. For propagation and proliferation of explants from DKW culture medium (Driver and Kuniyuki, 1984), 1 mg L^-1^ BA and 0.01 mg L^-1^ IBA and 30 g L^-1^ sucrose, as a suitable medium for the production of *in vitro* nodal shoots were used (Vahdati et al., 2004). Carrageenan was used as a gelling agent at a rate of 7 g L^-1^ in DKW medium. The pH of the culture medium was fixed at 5.7 before adding carrageenan and autoclave. Explants were kept in a growth room with a light period of 16 hours of light and 8 hours of darkness at a temperature of 25°C. To prepare the nodal shoot samples for light spectra treatments, micro shoots with terminal buds were first cut from 20 mm above it and cultured by placing 0.5 mm terminal micro shoots in the culture medium. The nodal shoot samples were about 15 mm in size and had no leaves. In this way, the same samples were obtained, and then these samples were transferred to the DKW base medium. To receive enough light, only two samples were cultured in each container. Six containers were used for each treatment and two explants were cultured per container. For light treatment, single nodes (20 ± 2 mm length) were obtained from *in vitro-*produced Persian walnut (cv. Chandler) and were used for the production of *in vitro* nodal shoots. The nodal shoot samples were cultured in 78-mm-diameter, 95-mm-height containers that contained DKW culture medium (50 mL) and placed in a controlled-environment chamber equipped with a 24-watt LED. Plantlets (nodal explants with leafy parts) were grown under photomixotrophic conditions (DKW medium supplemented with sucrose).

### 2.2 Lighting treatments

**Figure 1:**
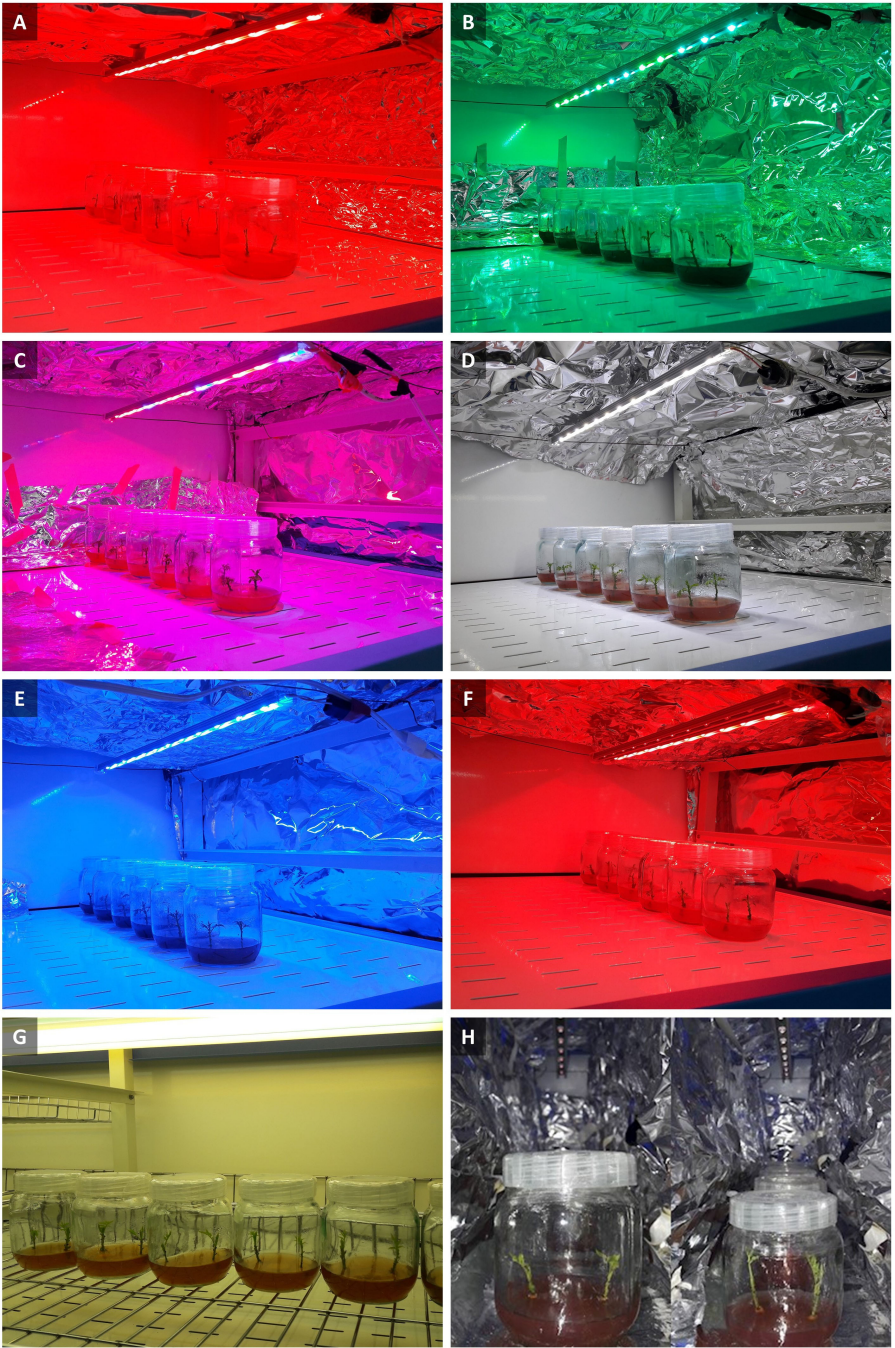
Walnut tissue culture vessels under different LED light qualities including: **(A)** Red (peak at 660 nm), **(B)** Green light (peak at 530 nm), **(C)** Combination of red and blue light (70: 30 ratio), **(D)** White light (400-700 nm), **(E)** Blue light (peak at 460 nm), **(F)** Combination of red and far-red light (70:30), **(G)** Fluorescent light (380-750 nm), **(H)** Far-red light (peak at 730 nm).

To investigate the effect of different spectra of light on *in vitro* explants of Persian walnut, an experiment in a completely randomized design with 8 treatments and six replications (two explants per replication) was designed. Phytotron supplemented with LED lights with a 20 cm vertical distance between the lights and culture vessels. explants of walnuts were grown for 28 days under different light spectra treatments including red (660 nm, **Figure 1A**), green (530 nm, **Figure 1B**), combination of red and blue (70: 30 ratio, **Figure 1C**), white (400-700 nm, **Figure 1D**), blue (460 nm, **Figure 1E**), combination of red and far-red light (70:30, **Figure 1F**), white (41% blue (400–500 nm), 39% intermediate (500–600 nm), and 20% red (600–700 nm), **Figure 1D**), fluorescent light as a control (380-750 nm, **Figure 1G**) and far-red light (730 nm, **Figure 1H**), under an intensity of 80 µmol m^-2^ s^-1^. Light intensity for eight light treatments was determined using a PAR-FluorPen device (FP 100-MAX, Photon Systems Instruments, Drasov, Czech Republic) and was considered similarly for all treatments. tissue-cultured walnut nodal shoots, which were cut from the terminal bud of the shoot and had the same size and no leaves, were placed in the Phytotron, which had the ability to adjust the temperature and photoperiod automatically, and had shelves with eight LED spectra (each shelf has a light treatment) were kept in a photoperiod of 16 hours of light and 8 hours of darkness. The wavelength of light treatments was measured by SpectroMaster (SEKONIC C-7000, Tokyo, Japan) in the range of 300-800 nm.

### 2.3 Measurement of morphological and growth characteristics

At the end of the experiment, following removing the explant samples from the culture containers (30 days after application of light treatments) vegetative traits such as stem length, internode length, stem diameter, callus length and width, leaf area, number of leaves and leaflets were measured in six replications for each treatment. Because the leaves of tissue-cultured plants undergo desiccation as soon as they take out of the containers, immediately stems and calluses are quickly separated so that they do not overlap and were placed on a white paper surface. The scale was placed next to them and quickly photographed by a professional camera (Canon EOS 90D Digital Camera With 18-135mm IS USM Lens), and then the parameters of stem length, callus length, and width, distance between nodes, and leaf area were measured by Digimizer software (v.5.4.9). Measurement by Digimizer software was as follows: first the measurement index was defined for the software and then the mentioned parameters were calculated for each treatment of a specific light spectra. Walnut Explants have leaves composed of Singular and large leaflets, and to calculate the number of leaves and leaflets, each compound leaf was counted as a leaf and each of its leaflets separately. Digital calipers were used to measure stem diameter.

Growth characteristics such as fresh and dry weight of leaves stems, and callus weights were measured after measuring leaf area using a digital scale in grams with six replications. After weighing, the samples were packed in paper bags and placed in an oven at 70°C for 48 h. After oven drying, their dry weight was determined.

### 2.4 Stomatal morphology

The effects of different light spectra on stomatal length, width, and density (i.e., number of stomata per unit of leaf area) together with pore length and pore width on *in vitro* explants of Persian walnut ‘Chandler’ were determined. Sampling was done 3 h after the start of the light period in the Phytotron, simultaneously under different light spectra, because this time is adequate for plants following experiencing the dark period to open stomata and reach a steady-state stomatal conductance (Seif et al., 2021). Due to the small size of tissue culture explant leaves, the sampling area was the entire leaf surface. The morphology of stomata was analyzed on the lower epidermis (adaxial surface) on the second lateral leaflets in an acropetally order in ten of the *in vitro*-nodal shoots per treatment (two explants per container with five replicates). Accordingly, under different light spectra, the adaxial leaf surface was painted with a thin layer of transparent nail polish. After a few minutes, the dried nail polish was detached from the leaf surface using transparent sticky tape. Sticky tapes with dried polish were mounted on the microscopic slides, and observations took place via a camera attached light microscope (Asayesh et al., 2017b). Images were taken by the Omax software (ver. 3.2, Omax Corp.). Finally, ImageJ (U.S. National Institutes of Health, Bethesda, MD; http://imagej.nih.gov/ij/) was used to measure the stomatal length, stomatal width, pore length, pore width, the ratio of stomatal length to stomatal width, and stomatal density. To measure stomatal traits, 100 stomata were used for each replication of light spectra treatment (Vahdati et al., 2017).

### 2.5 Chlorophyll fluorescence parameters

At the end of the period of light treatments, the maximum quantum efficiency of photosystem II (F_v_/F_m_) was measured using a FluorCam device (FluorCam FC 1000-H, Photon Systems Instruments, PSI, Drasov, Czech Republic). *in vitro* explants of Persian walnut (cv. Chandler) were placed in tissue culture jars in complete darkness for 20 min, and then the lid of the tissue culture jars was opened and the explants in the jar together with the tissue culture medium was used to measure slow induction of chlorophyll fluorescence in the Fluorcam photography section)without using the destructive method and separating the leaves). F_V_/F_M_ was calculated using a custom protocol in which F_0_ and F_m_ were recorded from the fluorescence data based on the protocol and two images were obtained from these two data series (Aliniaeifard and van Meeteren, 2014). F_v_ is expressed using the relationship F_v_=F_m_-F_0_. Finally, F_v_/F_m_ is obtained using the ratio (F_m_-F_0_)/Fm. Fluorcam software version 7 was used to analyze the photos related to each light treatment. At this stage, the calculations obtained from photographing each sample with the guide color spectra next to it show the level of plant photosynthesis health. The mentioned color guide from cool to warm color indicates the improvement of photosynthesis efficiency.

### 2.6 Leaf Photosynthetic pigment determinations

To measure the content of chlorophyll a, b, total chlorophyll and carotenoids in the leaves, 0.1 g of fresh leaf tissue was powdered with liquid nitrogen and homogenized in 2 mL of 96% ethanol. The resulting solution was centrifuged at 13000 rpm for 15 min. Since chlorophyll is light sensitive, extraction took place in a dark room (Bergsträsser et al., 2015). The obtained extract was subjected to reading on a spectrophotometer (Perkin Elmer Lambda 365 UV-Vis). Total chlorophyll and carotenoid contents were calculated (Lichtenthaler and Wellburn, 1983). To measure the amount of anthocyanin, 20 mg of fresh leaf tissue of *in vitro* explants of Persian walnut ‘Chandler’ were powdered by liquid nitrogen and homogenized with 2 mL of methanol acidified with 1% hydrochloric acid, and placed in an incubator at a temperature of 4 degrees for 24 hours. After one day, the resulting slurry was centrifuged at 13,000 rpm for 15 min, and then the anthocyanin in the supernatant was read at 530 and 657 nm with a spectrophotometer (Teng et al., 2005).

### 2.7 Determination of carbohydrates

The determination of soluble carbohydrates in the leaves of walnut tissue culture explants under different light spectra was determined by the Antron method using glucose as the standard. To do so, 0.05 g of fresh leaf tissue of *in vitro* explants of Persian walnut was powdered using liquid nitrogen, and five mL of 95% ethanol was added to it. Then the upper part of the solution was separated and the remaining sediments were washed again with five milliliters of 70% ethanol and the upper part was added to the previous solution. The extracted extract was centrifuged for 15 minutes at 4500 rpm and after separating the upper phase, the resulting alcoholic extract was used for carbohydrate measurement. To determine the total soluble sugars, 100 microliters of the resulting extract were taken and 3 mL of anthrone (150 mg of pure anthrone + 10 mL of 72% sulfuric acid) was added to it. Then it was placed in a water bath for 10 minutes to complete the reaction and make the extract colored. After the end of the Ben-Marie time, the falcons were quickly transferred to the refrigerator to cool down, and after cooling down, the absorbance at 625 nm wavelength was recorded using a spectrophotometer (van Doorn, 2012). To prepare the standard curve, pure glucose with concentrations of 0, 100, 200, 300, 400, and 500 mg L^-1^ used (McCready et al., 1950).

### 2.8 Statistical analysis

This study was conducted in a completely randomized design with eight treatments. Data analysis of variance was performed using SAS 9.4 software and the means were compared using the Duncan’s multiple range test at a probability level of 5%. The data related to stomatal morphology were evaluated by GraphPad Prism7 software (Graph Pad software, Inc., San Diego, CA), and a probability level of 5% (*P ≤ 0.05*) was considered to check the differences. For stomata morphological, physiological, and vegetative traits, data were subjected to ANOVA, and *P ≤ 0.05* was considered as not significant. For stomatal characteristics, data obtained from one leaf was considered not independent, and paired t-test was used to find significant differences (*P ≤ 0.05*) between the two groups.

## 3 Results

### 3.1 LED spectra improved the morphological characteristics of walnut explants compared to fluorescent light *in vitro*

**Figure 2:**
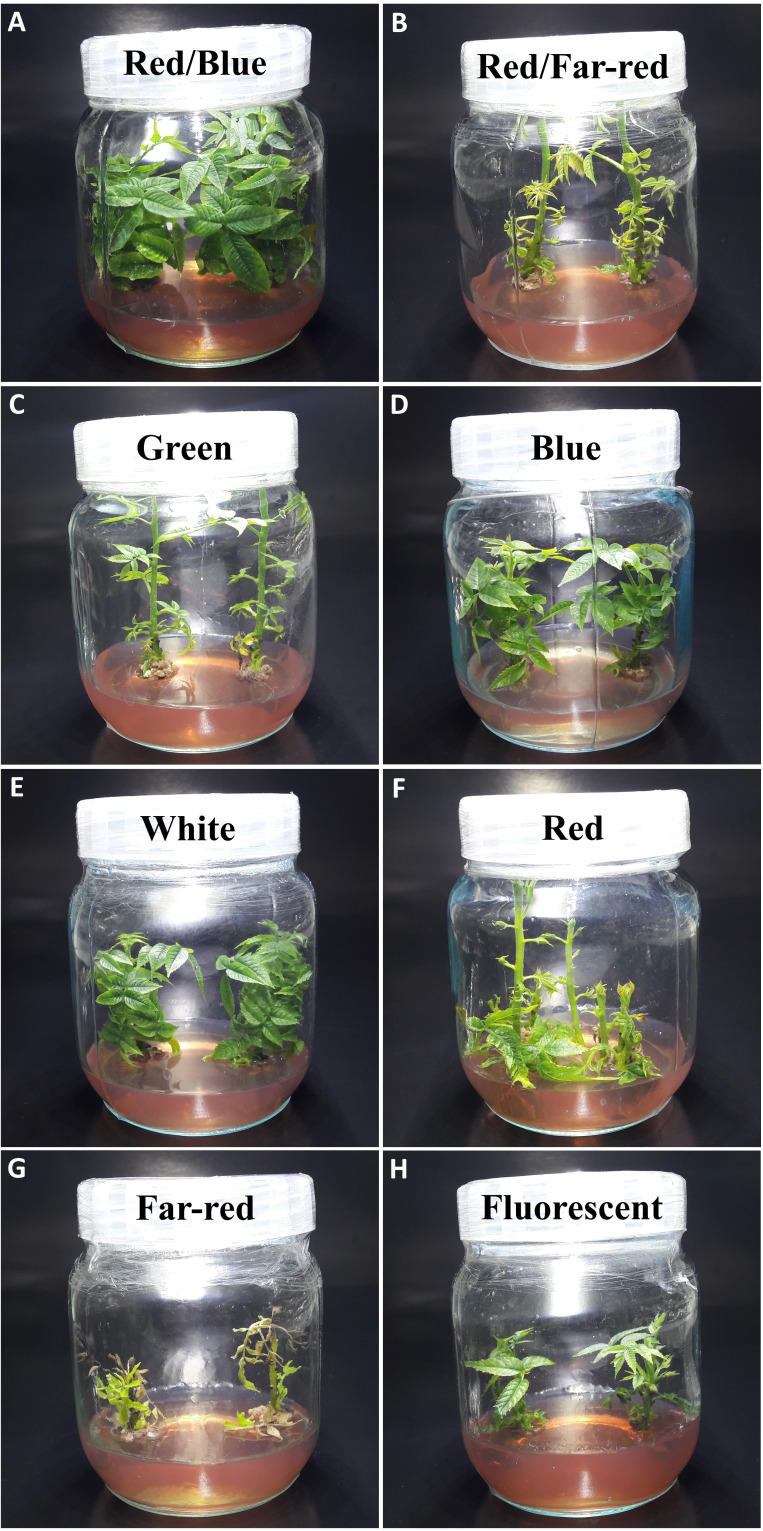
Morphology of *in vitro* explants of Persian walnut ‘Chandler’ grown under different light qualities including: **(A)** Combination of red and blue light (70:30), **(B)** a combination of red and far-red light (70:30), **(C)** green (wavelength peak at 530 nm), **(D)** blue (wavelength peak at 460 nm), **(E)** white light (400-700 nm), **(F)** red (wavelength peak at 660 nm), **(G)** Far-red light (wavelength peak at 730 nm), **(H)** fluorescent light (380-750 nm).

**Figure 3:**
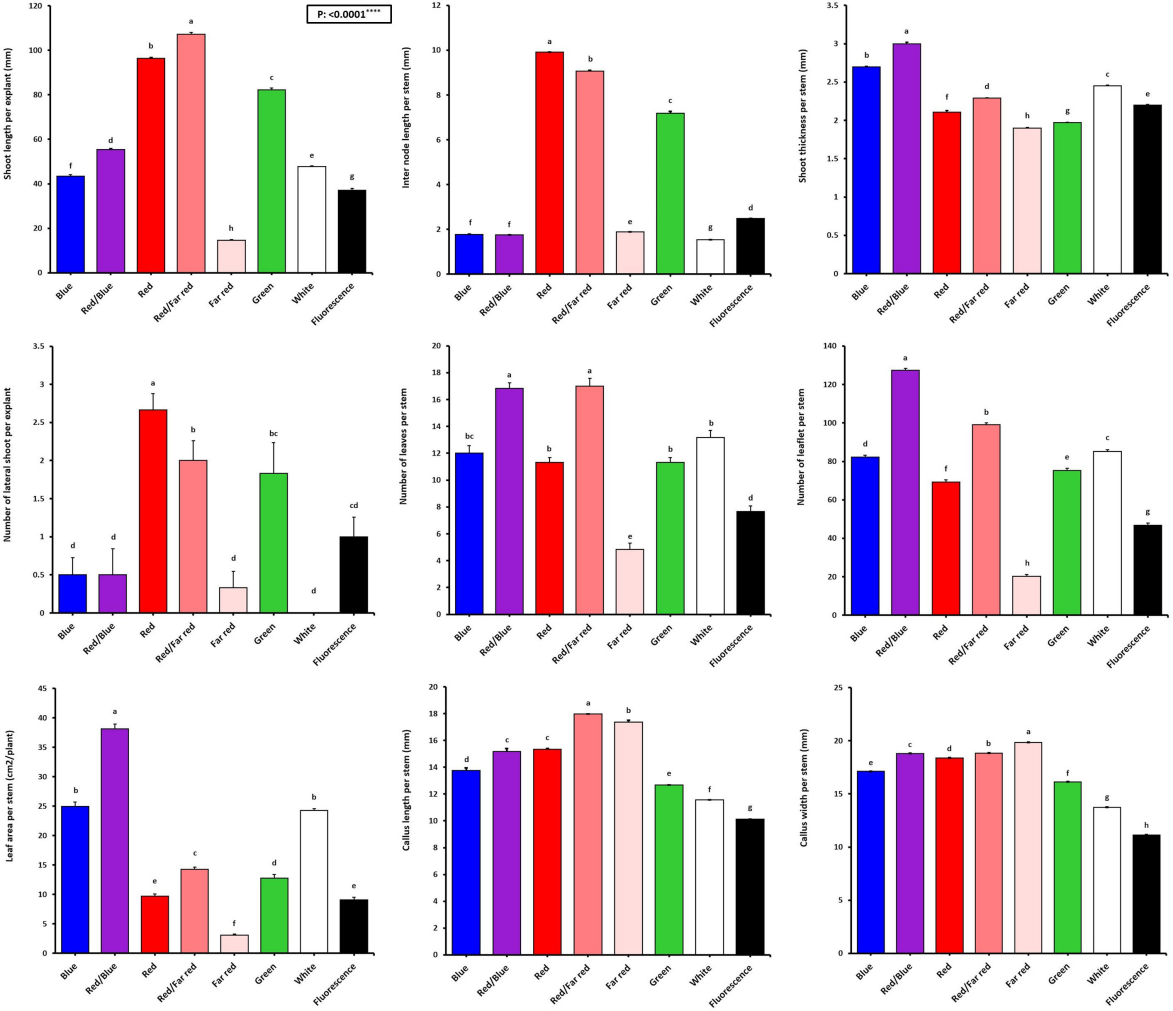
Vegetative characteristics of *in vitro* explants of Persian walnut ‘Chandler’ grown under different light qualities including: white light (700-400 nm), blue (wavelength peak at 460 nm), red (wavelength peak at 660 nm), far-red (wavelength peak at 730 nm), green (wavelength peak at 530 nm), a combination of red and blue light (70:30), a combination of red and far-red light (70:30) and a fluorescent light as a control (380-750 nm). Six explants per treatment were assessed. Bars represent SE. Values with different letters are significantly different at P < 0.05. Significance at the 0.0001 probability level is indicated by ****.

**Figure 4:**
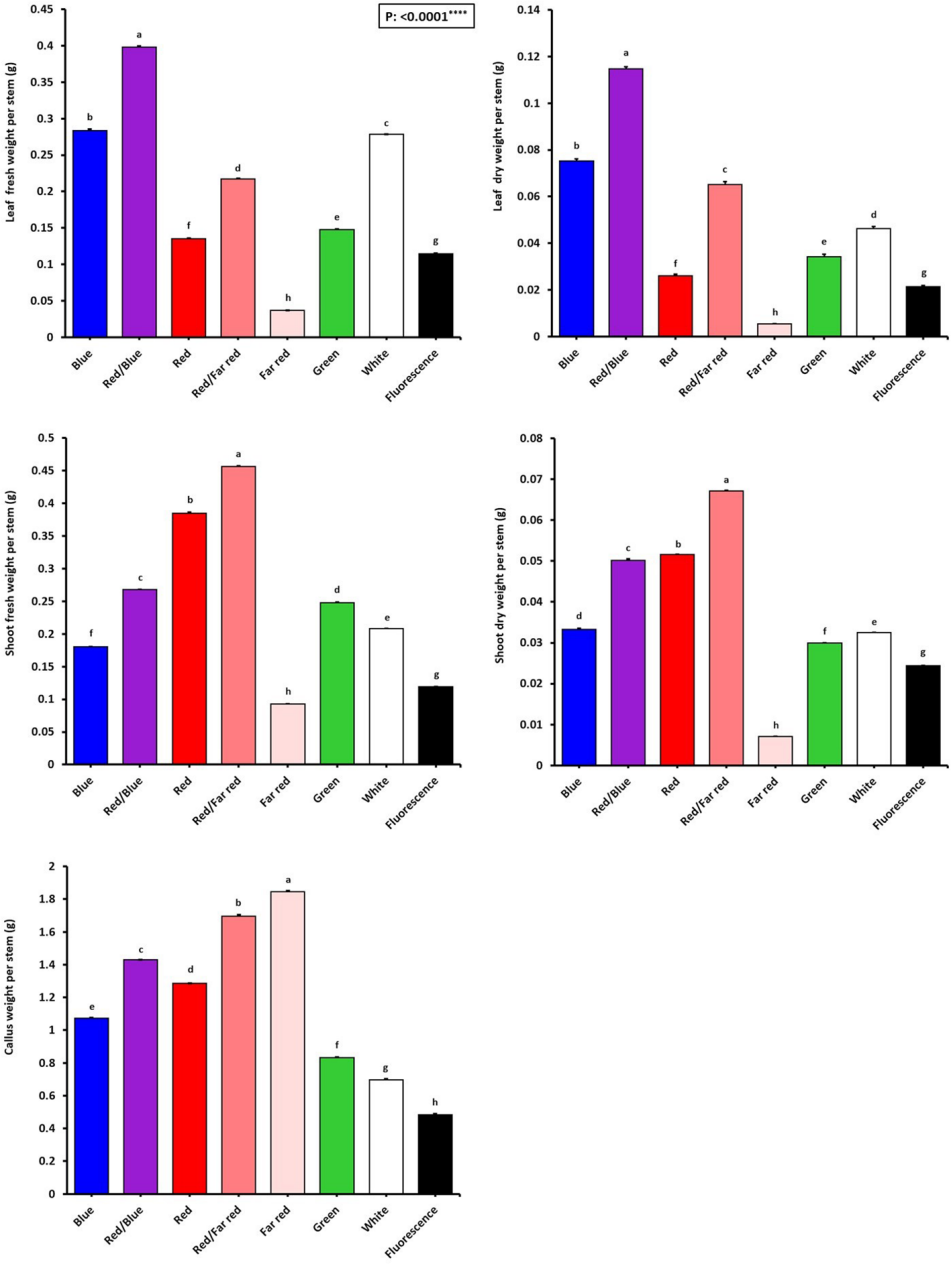
Growth characteristics of *in vitro* explants of Persian walnut ‘Chandler’ grown under different light qualities including: white light (400-700 nm), blue (wavelength peak at 460 nm), red (wavelength peak at 660 nm), far red (wavelength peak at 730 nm), green (wavelength peak at 530 nm), a combination of red and blue light (70:30), a combination of red and far-red light (70:30) and a fluorescent light as a control (380-750 nm). Six explants per treatment were assessed. Bars represent SE. Values with different letters are significantly different at P < 0.05. Significance at the 0.0001 probability level is indicated by ****.

**Figure 2** shows that the growth and morphological characteristics of the tissue cultures are highly influenced by the light spectra (**Figure 2**). The effect of light spectra treatments on all morphological characteristics of *in vitro* explants of Persian walnut was significant (*P<0.0001*). The highest stem length (107.22 mm) and the highest values ​​of shoot fresh and dry weights were recorded in samples exposed to a combination of red and far-red spectra. Far-red monochromatic light spectrum had negative effects on the stem length, shoots fresh and dry weights compared to their corresponding values under other light spectra. Indeed, explants did not grow under far-red light treatment. The combination of red and far-red LEDs increased plant height by 45.188%, stem fresh weight by 283.61%, and stem dry weight by 179.16% compared to their corresponding values under the fluorescent light (control). Under the single-color far-red LED, a decrease in stem length by 60.85%, stem fresh weight by 21.84% and stem dry weight by 70.83% in comparison with the control treatment was detected (**Figures 3**, **4**).

The number of lateral shoots and internode length were influenced by the light treatments (*P<0.0001*). Red monochromatic light spectrum caused the highest number of lateral shoots in walnut explants (2.66 numbers). the number of lateral shoots in red light-exposed samples increased by 166% compared to their number in samples exposed to fluorescent light. The tissue-cultured walnut nodal shoots did not produce lateral shoots under white LED, and the average number of lateral shoots under the fluorescent light was only one. The internode length was increased more than four times under the monochromatic red LED spectra treatment compared to the fluorescent light. The longest and shortest lengths of the internode (9.91 and 1.53 mm) were detected under the monochromatic red LED and white LED spectra, respectively (**Figure 3**).

Shoot thickness significantly differed among different LED light treatments (*P<0.0001*). The thickest shoot was observed in explants grown under the combination of blue and red LED spectra (2.99 mm), and the lowest shoot thickness was observed under the far-red LEDs (1.90 mm). The combination of blue and red LED spectra increased shoot thickness by 35.9% compared to the shoot thickness of walnut explants under fluorescent light (**Figure 3**).

### 3.2 Combination of blue and red LED spectra improved the growth traits of walnut explants *in vitro*

The effect of light spectral treatments on all growth characteristics of *in vitro* explants of Persian walnut (cv. Chandler) was significant (*P<0.0001*). Walnut tissue-cultured explants under a combination of blue and red LEDs showed the most expansive leaf area and the highest number of leaves than the explants under other light treatments. They also had the largest leaf area (38.15 cm^2^), number of leaflets (127.33), and fresh and dry weights. On the other hand, monochromatic red light restricted leaf emergence and area. Combination of red and blue LEDs in the growth chamber of *in vitro* explants of Persian walnut increased the leaf area by more than three times, the number of leaflets by almost two times, the fresh weight of leaves by more than two times, and the dry weight of leaves by more than four times compared to their corresponding values under the fluorescent light (control). Far-red monochromatic LED reduced the leaf area by 66.39%, the number of leaflets by 56.95%, the fresh weight of the leaf by 68.47% and the dry weight of the leaf by 4.287% in comparison with the explants in the control condition (**Figures 3**, **4**).

### 3.3 Far-red light induces callus formation in walnut explants *in vitro*

The highest amount of callus was observed under the far-red light (1.84 g). In comparison, the explants that were grown under fluorescent lights had the lowest callus weight (0.48 g) than the callus weight under other treatments. As a result of using far-red LED spectra in the walnut tissue culture growth room, the callus weight increased by 283.33% compared to its corresponding value under the fluorescent light. The largest callus width was also observed in the explants grown under far-red light treatment, which was increased by 78% compared to the fluorescent light. Furthermore, the length and width of the callus were the lowest in explants under fluorescent light (**Figures 3**, **4**).

### 3.4 Pigmentation influenced by *in vitro* environment light spectrum

**Figure 5:**
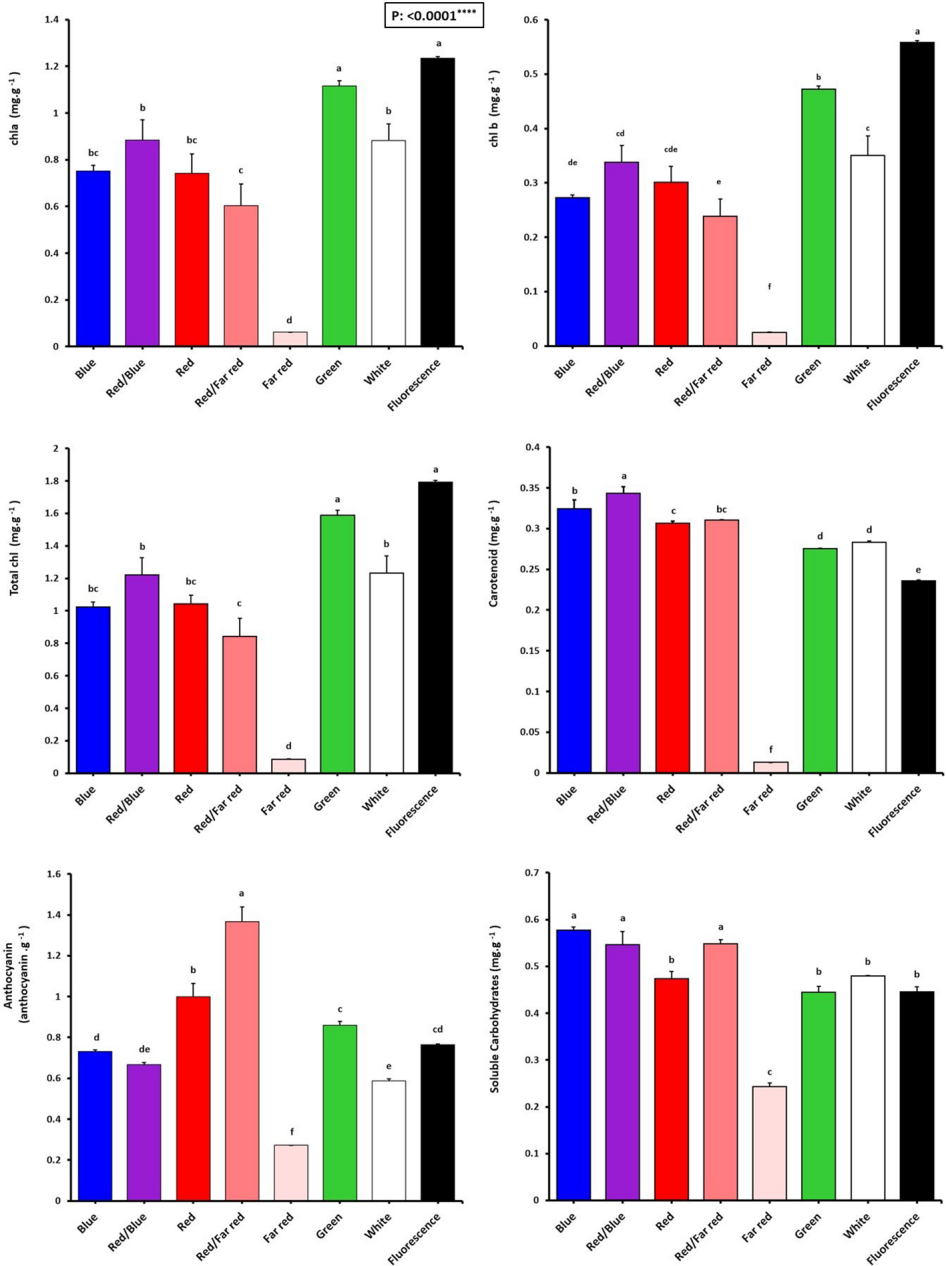
Pigment concentrations of *in vitro* explants of Persian walnut ‘Chandler’ grown under different light qualities including: white light (400-700 nm), blue (wavelength peak at 460 nm), red (wavelength peak at 660 nm), far-red (wavelength peak at 730 nm), green (wavelength peak at 530 nm), a combination of red and blue light (70:30), a combination of red and far-red light (70:30) and a fluorescent light as a control (380-750 nm). Six explants per treatment were assessed. Bars represent SE. Values with different letters are significantly different at P < 0.05. Significance at the 0.0001 probability level is indicated by ****.

The effect of light spectra on the biochemical characteristics of *Persian walnut in vitro explants* was significant (*P<0001*). Pigment concentration was analyzed to investigate the effects of different light spectra on pigment accumulation in the leaves of *in vitro* explants of Persian walnut ‘Chandler’. The light spectra significantly influenced all photosynthetic pigments (**Figure 5**). The chlorophyll pigments, carotenoids, and anthocyanins significantly decreased with the *in vitro* growth of walnut under far-red light.

The chlorophyll content (chlorophyll a, b, and total chlorophyll) of walnut explants under green LED and fluorescent light were higher than those under other light spectra. Instead, the far-red LED treatment showed the lowest amount of total chlorophyll, which was 95.1% lower than their content in explants under fluorescent light.

Carotenoid content was the highest in walnuts grown *in vitro* under a combination of blue and red LEDs, which was 47.82% higher than its content in the control treatment. Tissue cultured walnut micro-shoots under far-red LED treatment have the lowest carotenoid content, 94.34% lower than in the control treatment.

Anthocyanin content was the highest in walnut *in vitro*-explants under red and far-red combination, which was 78.94% compared to the anthocyanin content of the control (fluorescent light). The findings indicate that a combination of red and far-red spectra serves as an environmental cue for inducing anthocyanin production in the leaves of Persian walnut *in-vitro* explants (**Figure 5**).

### 3.5 Monochromatic blue LED together with red and far-red light spectra induced accumulation of soluble carbohydrates in walnut tissue cultured explants *in vitro*

The highest concentrations of soluble carbohydrates were detected in explants of Persian walnut grown *in vitro* under a monochromatic blue light spectrum, as well as the combination of red-light spectra with blue and far-red. On the other hand, soluble carbohydrates were significantly reduced by exposure to far-red LED spectra (**Figure 5**).

### 3.6 Blue light is important to maintain photosynthetic functionality in walnut explants *in vitro*

**Figure 6:**
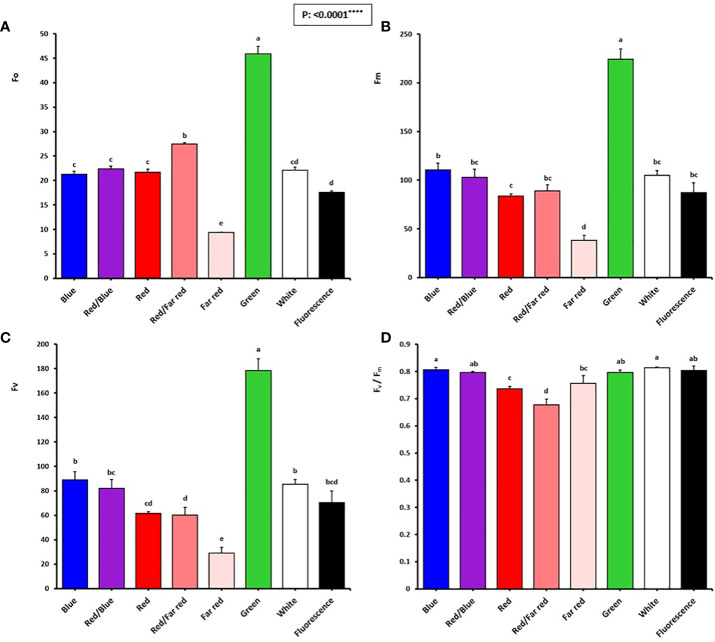
Minimum fluorescence intensity (F_o_; **A**), maximal fluorescence intensity (F_m_; **B**), variable fluorescence (f_v_: f_m_-f_o_; **C**), and maximum PSII efficiency (Fv/Fm; **D**) of *in vitro* explants of Persian walnut ‘Chandler’ grown under different light qualities including: white light (400-700 nm), blue (wavelength peak at 460 nm), red (wavelength peak at 660 nm), far-red (wavelength peak at 730 nm), green (wavelength peak at 530 nm), a combination of red and blue light (70:30), a combination of red and far-red light (70:30) and fluorescent light as a control (380-750 nm). Six explants per treatment were assessed. Bars represent SE. Values with different letters are significantly different at P < 0.05. Significance at the 0.0001 probability level is indicated by ****.

**Figure 7:**
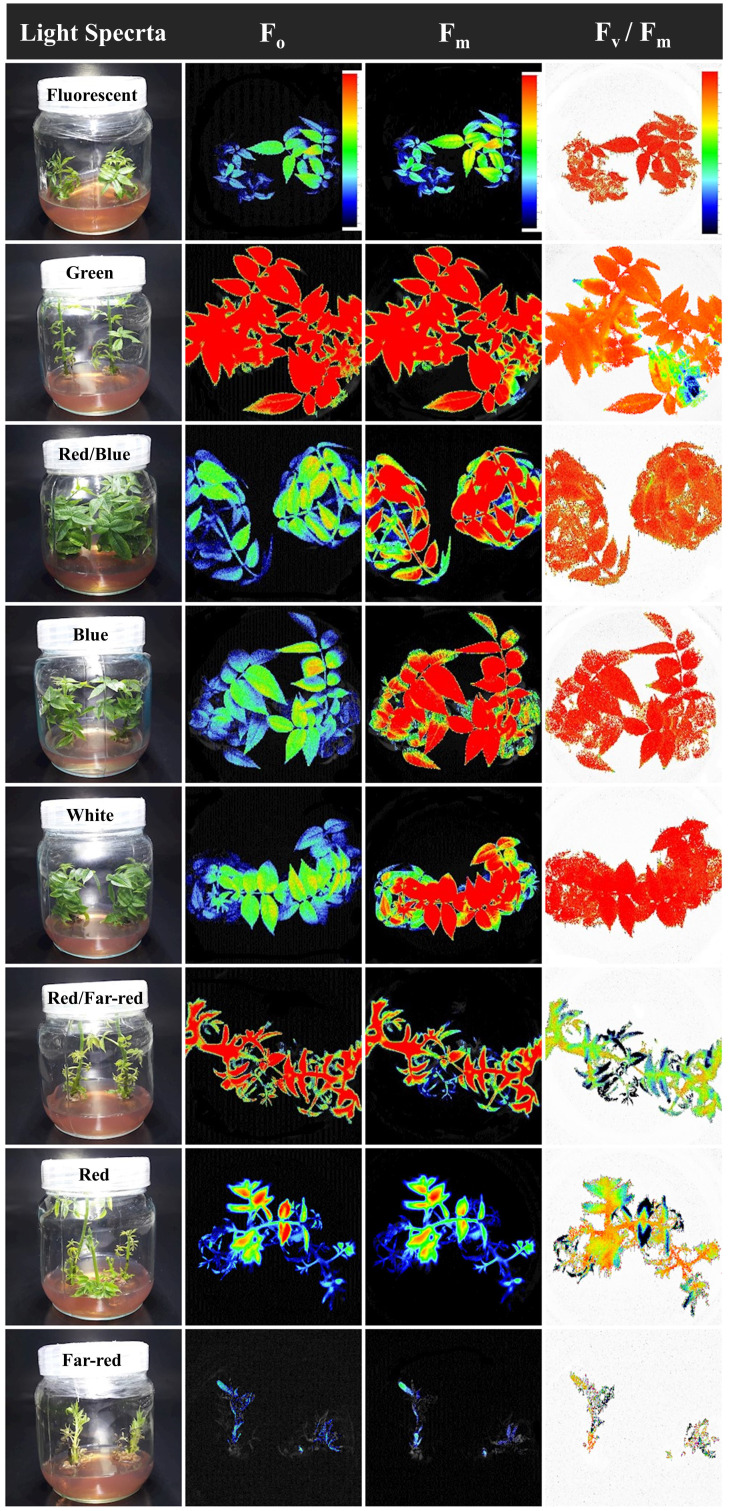
Pseudo-color images of F_0_, F_m_, and F_v_/F_m_ sampled from all canopy parts of walnut tissue culture explants exposed to different light qualities including: fluorescent (control), green, combination of blue and red, blue, white, combination of red and far-red, red and also far-red for 30 days. For the color scale presented in the first figure, warm colors (to the red) are indicative of higher values for the biophysical parameters of photosynthesis (F_0_, F_m_, F_v,_ and F_v_/F_m_), and cold colors (to the blue) are indicative of lower values for the biophysical parameters of photosynthesis.

The emission of chlorophyll fluorescence (photosynthesis biophysics) of *in vitro* explants of Persian walnut ‘Chandler’ was significantly influenced (*P<0.0001*) by a light spectrum of the *in vitro* environment. The energy content of different light spectra is absorbed by the chlorophyll pigments, which directly affects the electron transport chain of the photosynthetic apparatus. To assess the photosynthetic functionality, the effect of different light spectra during the growth of *in vitro* walnut was evaluated by tracking the spatial pattern of fluorescence emission through pseudo-color images of F_0_, F_m_, and maximum quantum yield of PSII (F_v_/F_m_) (**Figure 6**; equations in **Figure 7**).

In **Figure 7**, warm hues (such as red) indicate an increase in the biophysical parameters of photosynthesis (F_0_, F_m_, F_v_ and F_v_/F_m_), such as the green spectrum treatment, and cold hues, such as the red spectrum treatment, indicate a decrease in the biophysical parameters of photosynthesis.

The optimum quantum yield of PSII was studied by calculating different chlorophyll fluorescence parameters in the explant following dark adaptation. Among the light treatments, explants exposed to the green LED had the highest F_0_, F_m_, and F_v_. The lowest F_0_, F_m_, and F_v_ intensity were detected in explants exposed to far-red light.

The F_v_/F_m_ in tissue-cultured walnut nodal shoots (cv. Chandler) was significantly affected by different light treatments (*P<0.0001*). Tissue-cultured walnut leaves exposed to those lights contained a blue spectrum in their overall spectra, including white, blue, red, and blue, fluorescent together with green LEDs showed the highest F_v_/F_m_ value. These results indicate a decrease in the efficiency of photosynthetic quantum performance under the red and far-red lights, which decreased by 16.25% compared to fluorescent light treatment (**Figures 6**, **7**).

### 3.7 Blue light-induced the generation of big stomata, while red light generated small stomata in walnut explants *in vitro*

**Figure 8:**
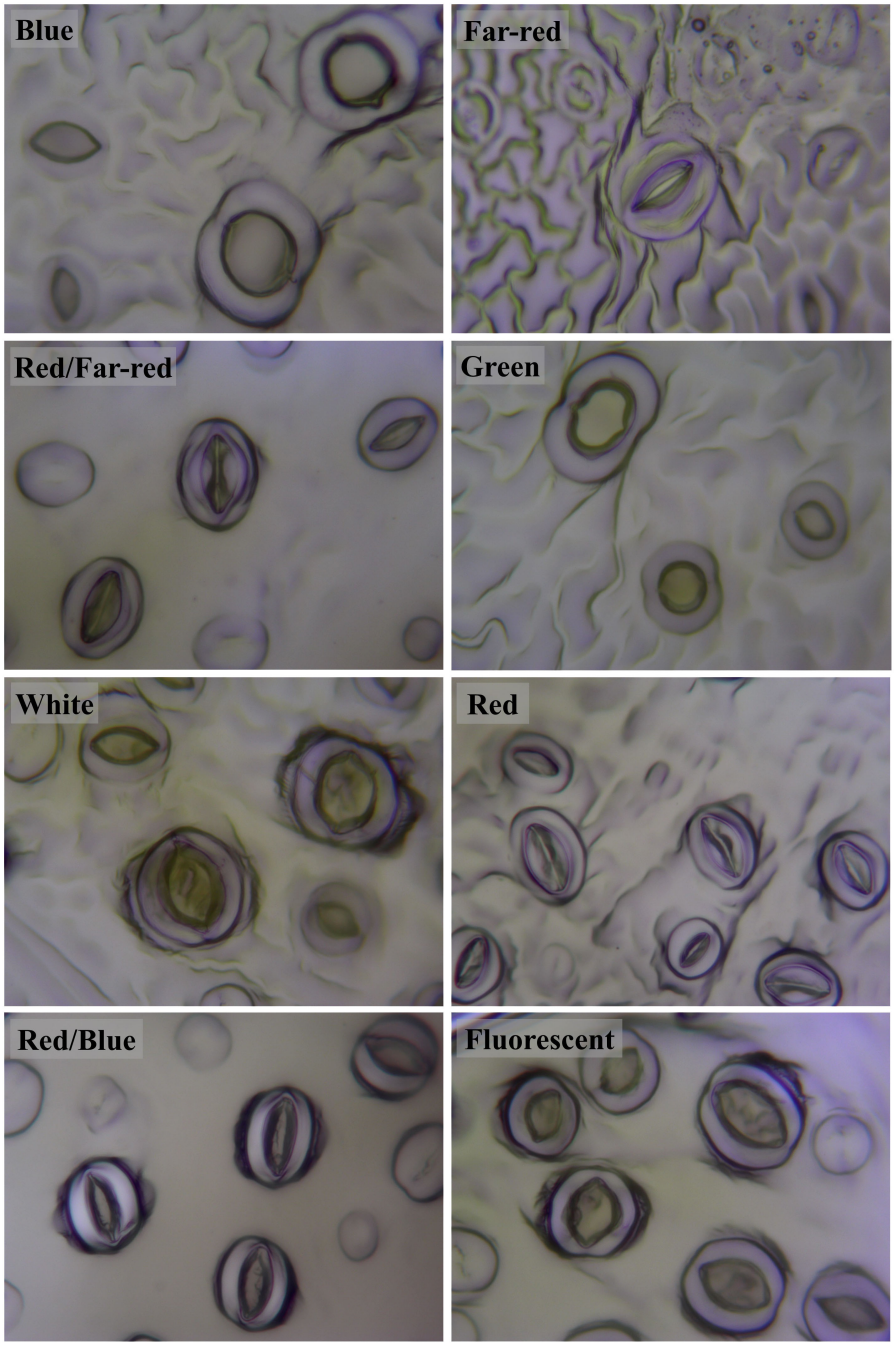
Stomata on abaxial surface of *in vitro* walnut (cv. Chandler) leaves developed under different light spectral conditions. Micropropagated shoots were grown in jars under different LED light qualities including: white light (400-700 nm), blue (wavelength peak at 460 nm), red (wavelength peak at 660 nm), far-red (wavelength peak at 730 nm), green (wavelength peak at 530 nm), a combination of red and blue light (70:30), a combination of red and far-red light (70:30) and a fluorescent light as a control (380-750 nm). The samples were incubated in a controlled environment chamber for 4 weeks at 25± 2°C and 16-h photoperiod.

**Figure 9:**
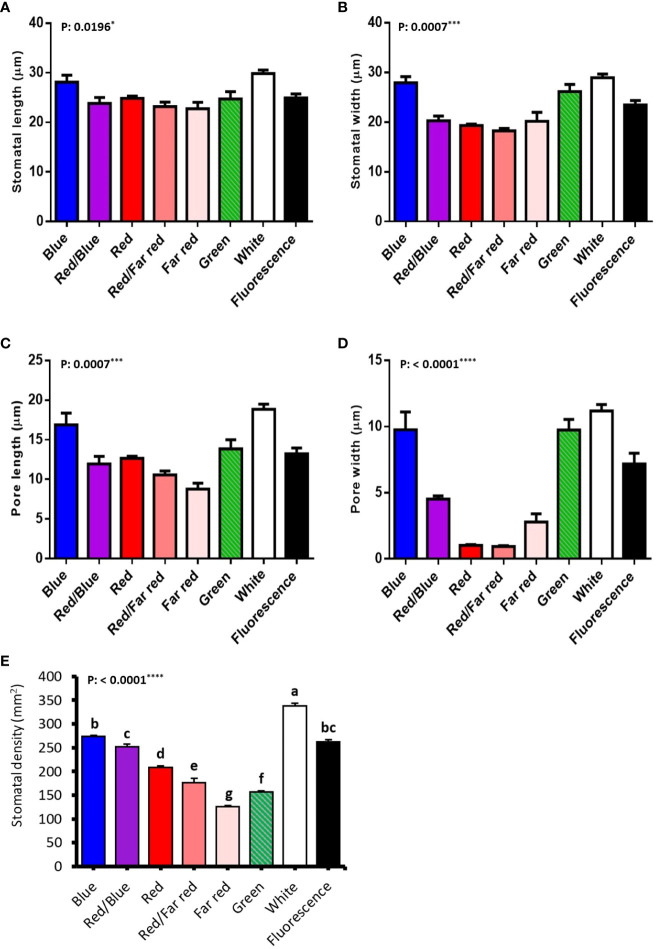
Stomatal anatomical characteristics (stomatal length **(A)**, stomatal width **(B)**, pore length **(C)**, pore width **(D)**, stomatal density **(E)** of *in vitro* explants of Persian walnut ‘Chandler’ grown under different light qualities including: white light (400-700 nm), blue (wavelength peak at 460 nm), red (wavelength peak at 660 nm), far red (wavelength peak at 730 nm), green (wavelength peak at 530 nm), a combination of red and blue light (70:30), a combination of red and far red light (70:30) and a fluorescent light as a control (380-750 nm). The data related to stomatal morphology characteristics were evaluated by GraphPad Prism7 software (Graph Pad software, Inc., San Diego, CA), and a probability level of five percent (P ≤ 0.05) was considered to check non-significance. For stomatal characteristics, data related to each leaf were considered independent. Significance at the 5% probability level is indicated by *, Significance at the 0.001 probability level is indicated by *** and Significance at the 0.0001 probability level is indicated by ****.

Stomatal size and pore dimensions in *in vitro* explants of Persian walnut ‘Chandler’ were considerably influenced by different light spectra (*P<0.0001*) (**Figure 8**; **Table 1**). The stomatal length and width and pore length and width in walnut tissue-cultured explants grown under white and blue LED spectra were more significant than other light treatments (**Figure 9**). The morphology of the stomata was influenced by the far-red and red LED spectra in both monochromatic mode and their combination. Monochromatic far-red LED spectra reduced the stomatal length and pore length compared to the stomata of samples under other light treatments (**Figures 9A, C**). Combination of red and far-red LED and monochromatic red spectra induced the generation of smaller stomata compared to the stomata of explants under other light treatments (**Figures 9B, D**).

**Table 1:** Variance analysis of stomatal characteristics on *in vitro* explants of Persian walnut ‘Chandler’ under light spectra treatments.

	Stomatal length	Stomatal width	Pore width	Pore length
Repeated measures ANOVA summary				
**Assume sphericity?**	No	No	No	No
**F**	4.967	15.23	42.96	12.85
**P value**	0.0196	0.0007	< 0.0001	0.0007
**P value summary**	*	***	****	***
**Statistically significant (P < 0.05)?**	Yes	Yes	Yes	Yes
**Geisser-Greenhouse’s epsilon**	0.4143	0.3493	0.3072	0.3968
**R square**	0.5539	0.792	0.9148	0.7626

****significance at the 0.0001 level, ***significance at the 0.001 level, and *significance at the 5% level.

The length of the stomata increased by 6.659 µm in the white LED spectra treatment compared to the far-red LED spectra treatment. Exposure to the white LED spectrum increased the stomatal width by 10.7 µm compared to the stomatal width of samples exposed to a red LED. The combination of red and far-red LED spectra reduced the width of the stomata by 9.65 µm compared to the stomatal width of samples grown under blue LED.

Under white LED, the pore length was 6.2, 8.2, 10.08 µm larger than the pore length of walnut explants exposed to red, a combination of red and far-red and Far-red monochromatic light LEDs. The pore width was reduced by 3.5 µm in the red LED spectra and 3.57 µm in the combined red and far-red LED spectra, compared to the blue and red combined LED spectra. The pore width increased by 8.803 µm under the green LED, 10.24 µm under the white LED, and 6.299 µm under the fluorescent light, compared to the pore width of the combination of red and far-red LEDs.

Explants treated with white LED spectra had the highest stomatal density, and those treated with far-red LED spectra had the lowest stomatal density. Using the white LED spectra in the tissue-cultured growth rooms increased the stomatal density by 28.88% compared to the fluorescent light. However, no significant difference was observed between blue and red combination LED spectra and fluorescent light. However, red LED, a combination of red and far-red, green and far-red decreased stomatal density.

**Figure 10:**
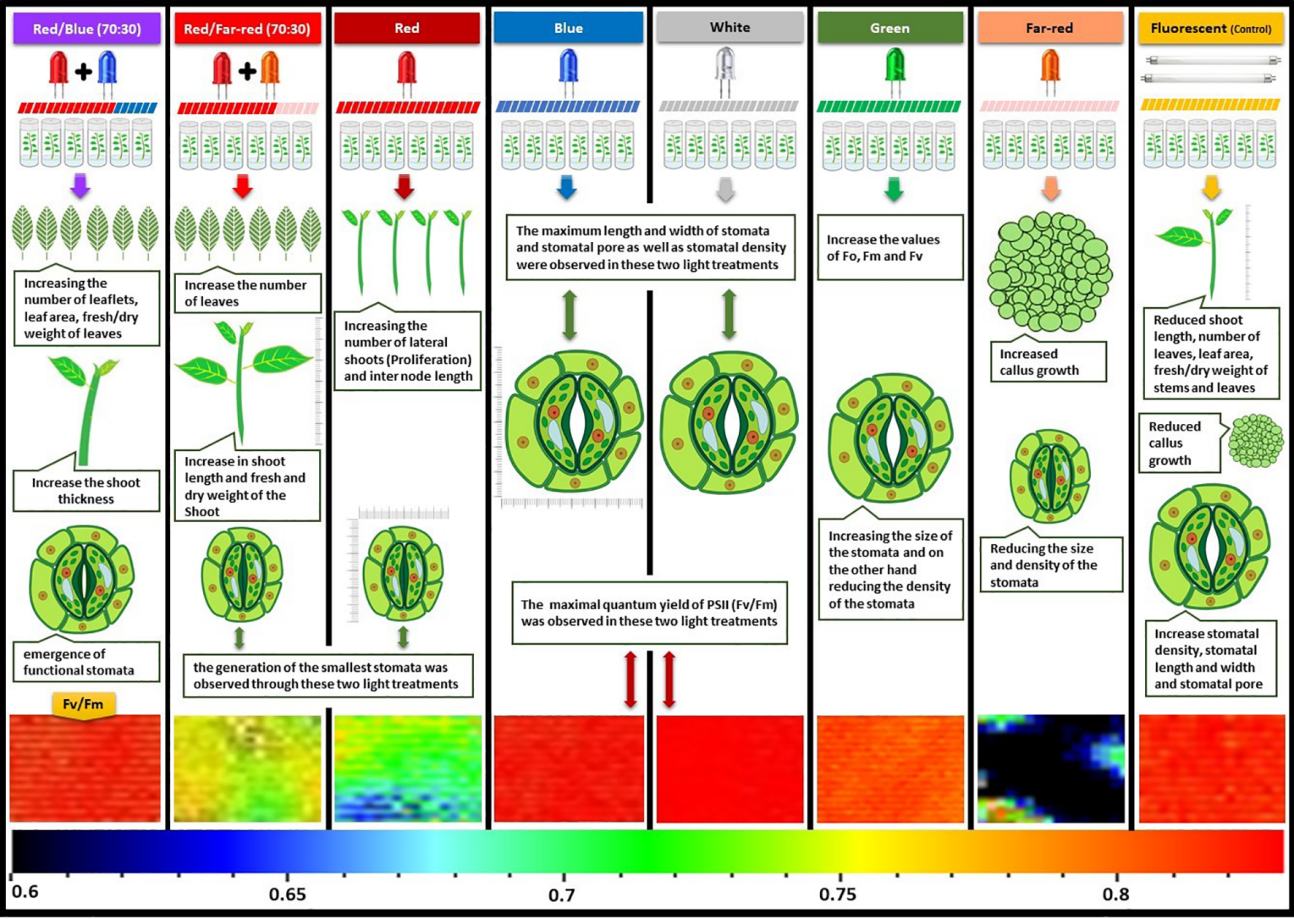
Graphical abstract about the effect of *in vitro* light spectrum on growth, stomatal function and photosynthetic characteristics of *in vitro* Persian walnut explants. For the color scale presented in the bottom of the figure, warm colors (to the red) are indicative of higher values for the maximum quantum efficiency of photosystem II (F_v_/F_m_), and cold colors (to the blue) are indicative of lower values for F_v_/F_m_.

**Figure 10** provides a graphical summary of the effect of light spectrum treatments on growth, stomatal function and photosynthetic characteristics of Iranian walnut explants.

## 4 Discussion

When producing Persian walnut *in vitro* explants, it is crucial to undergo hardening and acclimatization processes. This is due to the dysfunctional stomata of walnut *in vitro*-plantlets, resulting in excessive transpiration and heightened sensitivity to environments with increased evaporative demands and solar radiation (Chandra et al., 2010; Rathore et al., 2013; Manokari and Shekhawat, 2017). Plants are heterotrophic or mixotrophic under *in vitro* conditions, and their photosynthetic activity is limited (the carbon source is provided in the form of sugar). At the same time, they must grow in an autotrophic manner under *ex vitro* conditions.

Both quantity and quality of light are determinant factors for growth, regeneration and adaptation in tissues growing *in vitro* (Aalifar et al., 2019). Red light is usually increasing stem height and inter node length and rooting, but blue light is generally needed for the synthesis of chlorophyll and the development of stomata and leaf area expansion (Poudel et al., 2008; Li et al., 2010). In addition, blue light inhibits cell growth and regulates gene expression to prevent stem elongation (Lin et al., 2013). In the present study, the light spectra significantly affect the length of the generated shoots. In accordance with the obtained findings on walnut, red light increased the length of regenerated shoots in *in vitro* tissue culture samples of different plants species such as grapes (Heo et al., 2006), orchids (Chung et al., 2010), Chestnut (Park and Kim, 2010), and blueberries (Hung et al., 2016).

In the proliferation stage, when the length of the plant and the internode length increase, more explants are taken from the plantlet, and it increases the production in commercial tissue culture, which and also occurred in the present study. When walnut *in vitro*-explants were grown under red and far-red light spectra and monochromatic red-light spectrum, a significant increase in stem height and internode length was observed. The increase in internode length under the red and far-red spectra is due to increased cell length and cell division. GA turnover is higher in tissues exposed to far-red light (Beall et al., 1996). Both cell division and elongation contribute to the elongation response of plant internodes to red light, and these processes are associated with increased GA levels. These results indicate that GA signaling in walnut *in vitro*-explants is required for red and far-red light-induced shoot elongation.

The walnut tree is considered one of the most recalcitrant species, thereby presenting difficulties regarding its proliferation rate. Therefore, for proliferation in *in vitro* conditions, it is necessary to define the optimal environmental conditions for its proliferation (Sota et al., 2023). In the commercial tissue culture of walnut, the number of lateral shoots grown from one explant is very important. In the present study, we achieved this by using red LEDs without using chemical hormones, increase in the shoot length in plant tissue culture led to the production of more explants in the proliferation stage of the explants and increased the proliferation rate. The number of lateral shoots (proliferation rate) under the red LED spectra increased by 166% compared to the fluorescent light. This shows that red LED positively affects the formation of lateral shoots in tissue-cultured walnut nodal shoots. Similarly, in the studies conducted in the tissue culture plantlet of Stevia (Ramírez-Mosqueda et al., 2017) and banana, the red LED spectra increased the number of shoots in each explant (Trivedi and Sengar, 2017). In contrast, the combination of blue and red LED spectra has been reported to produce a high number of shoots per explant in Canola and Dendrobium (Lin et al., 2011; Lin et al., 2013).

In the present study, combining blue and red LED spectra and blue LED alone on tissue-cultured walnut nodal shoots increased the shoot thickness by 35.9% compared to the fluorescent light, consistent with the results obtained in *Gossypium hirsutum* (Li et al., 2010). However, the blue LED spectra increased the shoot thickness of tissue cultured cotton plantlets. Elongation by far-red light is due to the activation of shade avoidance response by far-red light and suppression of this process by blue light. Red light spectrum induces the levels of plant growth regulators, especially endogenous gibberellins, which play an essential role in cell elongation by stimulating mitosis in the apical and subapical meristem, which can lead to long stems and reduced diameter of the explants. On the other hand, the blue spectrum creates shorter shoots with larger diameters (Manivannan et al., 2021).

Appropriate leaf growth from regenerated shoots indicates a stable and tolerant growth response in *in vitro* conditions. LED spectra in tissue cultured walnut micro-shoots significantly affected leaf area, number of leaves, and fresh and dry weight of leaves. In the conditions where both blue and red lights were used, the growth of explants increased, and the red and blue LED spectra in different combinations improved the growth of leaves (number of leaves, leaf area, and leaf length). These findings are in line with those reported in tomato (Yousef et al., 2021), grape (Kurilčik et al., 2007), doritaenopsis (Shin et al., 2008), poplar (Kwon et al., 2015), potato (Ma et al., 2015), stevia (Ramírez-Mosqueda et al., 2017) and sugarcane (de Araújo Silva et al., 2016). The red spectrum prevents leaf expansion, resulting in smaller leaf area (Tan et al., 2022). On the other hand, blue spectrum, by increasing the ability to expand the cell wall in plant cells, increases the leaf area (**Figure 6**). In the current experiment, the combination of blue and red spectra increased the leaf area and the number of leaves and leaflets compared to other light treatments (**Figure 6**). Blue monochromatic LED spectra induced leaf formation in tissue cultured cotton plantlets (Li et al., 2010), while regenerated shoots of *Protea cynaroides* tissue culture explants responded to leaf development under red LED spectra (Wu and Lin, 2012). The use of far-red LED spectra in combination with other LED spectra improved leaf growth in Oncidium (Chung et al., 2010) and chestnut (Park and Kim, 2010) explants, which is consistent with the findings of the present research.

In our experiment, according to the results obtained from the treatments of different light spectra in tissue-cultured walnut nodal shoots, the highest amount of leaf fresh and dry weight was observed in explants grown under combined of blue and red LED spectra. The red spectrum plays an essential role in controlling chloroplast function, and stem and petiole growth (Johkan et al., 2010; Savvides et al., 2012). Plantlets that are placed under blue light have higher stomatal conductance, and chlorophyll a/b, better photosystem activity and photosynthetic electron transport capacity, higher levels of ribose-1, 5- bisphosphate carboxylase/oxygenase (Rubisco) activity and expression of genes related to Calvin cycle than those plants grown under red light (Muneer et al., 2014; Yang et al., 2018; Li et al., 2020). More significant biomass production results from the rapid expansion of leaf area or increase in the rate of photosynthesis per unit of leaf area, or both (Ohtake et al., 2018). Previous studies showed that the red LED spectra increase the fresh and dry weight of the stem in the Dendrobium tissue culture plant (Lin et al., 2011), which are confirmed in walnut *in vitro*-explants as well. In Yarrow tissue culture plantlets (Alvarenga et al., 2015), an increase in fresh and dry weight was observed under monochromatic red and blue LED spectra. Biomass was increased in regenerated plants of miniature coral (Dewir et al., 2006) and cotton (Li et al., 2010) under monochromatic and combined LED spectra treatments.

Chlorophylls play a significant role in photosynthesis. They have not so much absorption of light in the region of 450-550 nm, which is within the spectra of the sun’s radiation to the earth’s surface. This range is precisely the region where carotenoids absorb light strongly. Carotenoids can transfer this stimulated energy to chlorophylls and make it available to the plant to provide energy for photosynthesis. This energy transfer reaction allows carotenoids to act as additional light- absorbing pigments and expand the spectral range over which light can support photosynthesis (Green and Parson, 2003; Fromme, 2008). However, the reason that carotenoids are essential for photosynthesis is not because of their role in light absorbing, but because of their ability to prevent photo-damage under high light conditions (Green et al., 2003). In the present experiment, using the combined blue and red LED spectra on walnut tissue cultured samples, the highest amount of carotenoids was observed, which increased by 47.82% compared to the samples under fluorescent light. Accordingly in banana tissue cultured plantlets (Trivedi and Sengar, 2017), Stevia and Doritaenopsis (Ramírez-Mosqueda et al., 2017), the combined blue and red LED spectra increases the carotenoids concentration, which is consistent with the obtained results of present study.

It is well known that anthocyanin acts as a filter for light stress (Agati et al., 2021). In general, anthocyanin accumulation occurs due to long-term exposure of the plant to red and far-red light mediated by phytochrome. In contrast, the response to blue and ultraviolet light is created by cryptochrome or UV-B photoreceptors (Wang et al., 2012). Light plays a role in the activation of various enzymes involved in anthocyanin biosynthesis, especially phenylalanine ammonia lyase (PAL), which is a key enzyme in the anthocyanin biosynthesis pathway, and Chalcone synthase (CHS) (Simões et al., 2012). Light-induced anthocyanin accumulation is regulated through the activation of transcription factors (Nakatsuka et al., 2009). It has been shown in several studies that light radiation also has an important effect on anthocyanin production in *in vitro* cultured samples (Takeda, 1990; Galbiati et al., 1994; Guo et al., 2007). In the present experiment, as a result of using the combined red and far-red LED spectra, the amount of anthocyanin increased by 78.94% compared to the fluorescent light, which is consistent with the studies in which the red LED spectra increased anthocyanin (Lee et al., 2010; Bayat et al., 2018).

Carbohydrates are the final product of photosynthesis and the essential material for the growth and development of plants (Lin et al., 2013). The light source regulates carbohydrate metabolism in plants and hence affects plant growth (Lefsrud et al., 2008). Previously, it was reported that red light is the most effective light source for the accumulation of soluble carbohydrates (Li et al., 2012). Red and blue light causes the production of more photosynthetic products in plants. The combination of blue and red light also increases these compounds in plants (Xu et al., 2014). In the present experiment, the highest amount of soluble carbohydrates was observed in walnut *in vitro*-explants grown under the treatment of blue LED spectra, red and far-red and red and blue light combinations. the ratio of red and blue LED spectra can affect biochemical compounds, such as soluble carbohydrate, starch and amino acids. It has been found that light source, especially red and far-red light, affects carbohydrate accumulation (Damayanthi Ranwala et al., 2002). In an experiment conducted in date tissue culture plants, blue and red combined LED spectra increased soluble carbohydrates (Al-Mayahi, 2016). Furthermore, a combination of blue and red spectrum, monochromatic blue, and a combination of red and far red spectrum increased soluble carbohydrates, which is consistent with the results obtained in the present study (Damayanthi Ranwala et al., 2002).

Plants absorb light; part of the light is used for photochemical products, part is re-emitted as chlorophyll fluorescence, and the other part is released as heat, which is defined as the fluorescence of chlorophyll a (Schreiber, 1983). The emitted fluorescence has been widely used in previous studies for investigation of photosynthesis apparatus in response to different lighting conditions (Aliniaeifard et al., 2018; Ghorbanzadeh et al., 2021; Sousaraei et al., 2021; Ashrostaghi et al., 2022; Moosavi-Nezhad et al., 2022). Photosynthetic performance of walnut explants maintained by blue light and downregulated by red light in the present study. The high F_0_ indicates the lack of proper functioning of the photosystems and closure of reaction centers, the highest amount of F_0_ was detected in the samples exposed to green LED lights, which increased by 174.08% compared to the fluorescent treatment. When photosynthetic reactions are carried out slowly, the fluorescence intensity of chlorophyll is low, but in response to any disturbance in photosynthesis, the fluorescence intensity increases significantly (Lichtenthaler and Rinderle, 1988). The increase in F_0_ can be due to the inhibition of the reaction centers of photosystem II, which inhibits the transfer of electrons from QA to QB and decreases light trapping function in photosystem II (Falqueto et al., 2017). The reason for the increase in F_0_ is damage to the D1 protein attached to photosystem II (Falqueto et al., 2017).

F_v_/F_m_ is the maximum photochemical quantum yield in photosystem II, which is obtained from the difference between maximum fluorescence and minimum fluorescence over maximum fluorescence (Björkman and Demmig, 1987). A change in the amount of each parameter results in a change in the value of F_v_/F_m_. The increase in F_v_/F_m_ indicates the increase in photochemical efficiency of photosystem II (Guidi et al., 2019). Plants absorb blue and red-light spectra more than green and yellow light spectra (Wang et al., 2022). Different absorption for different lights may partially explain the difference in CO_2_ absorption of plants growing under different light spectra (Aalifar et al., 2020a; Aalifar et al., 2020b; Javadi Asayesh et al., 2021). In the present experiment, lighting environment containing red light alone or with far-red downregulated the photosynthesis functionality. Therefore, white and blue light spectra improve the efficiency of light energy conversion and increases energy accumulation for carbon absorption in carbon reactions. In a similar study, orchids had lower F_v_/F_m_ values under monochromatic red light (0% blue) than treatments with more blue light (Ouzounis et al., 2015). In other experiments, the highest value of F_v_/F_m_ was detected under white and blue light spectra (Guo et al., 2007; Chen et al., 2014; Wang et al., 2015). In a study, the highest value of F_v_/F_m_ was observed under blue LED spectrum and the lowest value was observed under red LED spectrum (Xiaoying et al., 2012; Aliniaeifard et al., 2018; Seif et al., 2021), which is in line with the results of the present research.

Stomatal morphology depends on the lighting environment (Ghorbanzadeh et al., 2021; Seif et al., 2021). In the present experiment, monochromatic red and far-red light and their combination (for example, the absence of blue light) led to the generation of small stomata with narrow apertures. Instead, light spectra treatments with increased percentages of blue light, i.e., blue and white light, induced generation of large stomata with wide apertures. Our results are consistent with the results of another study in which white and blue LED spectra increased the stomatal size with open pores and the red-light spectrum showed the opposite effects (Aalifar et al., 2020a; Aalifar et al., 2020b; Seif et al., 2021). The inductive role of blue light in the promotion of stomatal size and aperture has been suggested previously (XiaoYing et al., 2011; Savvides et al., 2012). In the present study, the growth of *in vitro* explants of Persian walnut under white LED spectra treatment resulted in the highest stomatal density compared to other light treatments, which is consistent with other studies in which white LED spectra increased stomatal density (do Nascimento Vieira et al., 2015; Seif et al., 2021). Previous research also shows that broad light spectra result in higher stomatal density compared to monochromatic light spectra (Lee et al., 2007; Savvides et al., 2012). Both blue and red light spectra can induce the opening of the stomata, but the role of red light in the beginning of the stomata is indirect. The effect of blue light on the opening of the stomata is not dependent on the mesophyll, and this light can directly induce swelling of the guard cells of the stomata and, as a result, open them. Through its receptors, blue light can now cause hyperpolarization in guard cells and induce the penetration of ions, resulting in the opening of plant stomata (Kinoshita and Hayashi, 2011), while the role of red light in space of the stomata is indirect and through the photosynthesis of mesophyll cells and chloroplasts (Olsen et al., 2002; Suetsugu et al., 2014).

## 5 Conclusion

The growth and development of Persian walnut ‘Chandler’ explants *in vitro* can be affected by different light spectra, which also have an impact on their photosynthetic functionality and stomatal characteristics. When using LED technology and controlling light quality, the growth and proliferation of *in-vitro* explants can be increased. Using specific light spectra with LEDs can be effective for the vigorous growth of tissue-cultured walnut nodal shoots with proper photosynthetic functionality and stomata. The combination of blue and red LED spectra proved to be particularly beneficial for the growth and vegetative characteristics of walnut explants *in vitro*, as well as for the production of carotenoids. The largest and smallest sizes of stomata were detected under blue-containing and red-containing spectra, respectively, while the combination of blue and red LEDs resulted in intermediate sizes of stomata. Additionally, red light downregulated the photosynthetic functionality of the walnut tissue cultured samples, while blue light maintained it. Therefore, using a combination of blue and red LEDs during *in vitro* culture of walnuts can be a useful tool to improve growth and to induce functional stomata and photosynthesis in *in vitro* walnut explants.

## Data availability statement

The original contributions presented in the study are included in the article/supplementary material. Further inquiries can be directed to the corresponding authors.

## Author contributions

SS: Conceptualization, Investigation, Project administration, Supervision, Validation, Writing - review & editing. KV: Data curation, Methodology, Software, Visualization, Writing - original draft. SA: Conceptualization, Investigation, Project administration, Resources, Supervision, Validation, Writing - review & editing. SS: Conceptualization, Investigation, Software, Validation, Writing - review & editing. SD: Conceptualization, Investigation, Validation, Writing - review & editing. MD: Software, Visualization, Writing - original draft. NG: Funding acquisition, Resources, Validation, Writing - review & editing.
